# Effects of Human and Porcine Adipose Extracellular Matrices Decellularized by Enzymatic or Chemical Methods on Macrophage Polarization and Immunocompetence

**DOI:** 10.3390/ijms22083847

**Published:** 2021-04-08

**Authors:** Mónica Cicuéndez, Laura Casarrubios, María José Feito, Iratxe Madarieta, Nerea Garcia-Urkia, Olatz Murua, Beatriz Olalde, Nerea Briz, Rosalía Diez-Orejas, María Teresa Portolés

**Affiliations:** 1 Departamento de Bioquímica y Biología Molecular, Facultad de Ciencias Químicas, Universidad Complutense de Madrid, Instituto de Investigación Sanitaria del Hospital Clínico San Carlos (IdISSC), 28040 Madrid, Spain; mcicuendez@ucm.es (M.C.); laura.casarrubios.molina@gmail.com (L.C.); mjfeito@ucm.es (M.J.F.); 2 TECNALIA, Basque Research and Technology Alliance (BRTA), E20009 Donostia-San Sebastian, Spain; iratxe.madarieta@tecnalia.com (I.M.); nerea.garcia@tecnalia.com (N.G.-U.); olatz.murua@tecnalia.com (O.M.); nerea.briz@tecnalia.com (N.B.); 3 Departamento de Microbiología y Parasitología, Facultad de Farmacia, Universidad Complutense de Madrid, 28040 Madrid, Spain; 4 CIBER de Bioingeniería, Biomateriales y Nanomedicina, CIBER-BBN, 28040 Madrid, Spain

**Keywords:** extracellular matrix, decellularization, macrophage, immunocompetence, phagocytosis

## Abstract

The decellularized extracellular matrix (ECM) obtained from human and porcine adipose tissue (AT) is currently used to prepare regenerative medicine bio-scaffolds. However, the influence of these natural biomaterials on host immune response is not yet deeply understood. Since macrophages play a key role in the inflammation/healing processes due to their high functional plasticity between M1 and M2 phenotypes, the evaluation of their response to decellularized ECM is mandatory. It is also necessary to analyze the immunocompetence of macrophages after contact with decellularized ECM materials to assess their functional role in a possible infection scenario. In this work, we studied the effect of four decellularized adipose matrices (DAMs) obtained from human and porcine AT by enzymatic or chemical methods on macrophage phenotypes and fungal phagocytosis. First, a thorough biochemical characterization of these biomaterials by quantification of remnant DNA, lipids, and proteins was performed, thus indicating the efficiency and reliability of both methods. The proteomic analysis evidenced that some proteins are differentially preserved depending on both the AT origin and the decellularization method employed. After exposure to the four DAMs, specific markers of M1 proinflammatory and M2 anti-inflammatory macrophages were analyzed. Porcine DAMs favor the M2 phenotype, independently of the decellularization method employed. Finally, a sensitive fungal phagocytosis assay allowed us to relate the macrophage phagocytosis capability with specific proteins differentially preserved in certain DAMs. The results obtained in this study highlight the close relationship between the ECM biochemical composition and the macrophage’s functional role.

## 1. Introduction

The decellularized extracellular matrix (ECM) obtained from different tissues is currently being used for the preparation of biomaterials designed for regenerative medicine [1]. The components of ECM are mainly collagen, elastin, fibronectin, laminins, proteoglycans, glycosaminoglycans, and glycoproteins [2]. All these molecules constitute a framework for the cells and regulate biochemical and biomechanical processes involved in cell morphogenesis and differentiation [3]. Thus, the cell–ECM interactions control different signaling pathways modulating the gene transcription, cell phenotype, and specific responses that are essentials for tissue homeostasis [4]. In this context, ECM-based biomaterials are proposed to promote rapid healing at the implant site and reduce foreign body reactions [5]. However, the ability of these natural biomaterials to modulate host immune reactions is not yet known accurately. The previous removal of cells, named decellularization, is a very important phase in the preparation of ECM-based bio-scaffolds to avoid undesired immune responses and implant rejection [6]. In recent years, several studies have focused on the decellularization process of human and porcine adipose tissue (AT) [7]. This tissue has high potential as an abundant source of ECM, thus representing an ideal material for preparing scaffolds designed for the growth, differentiation, and phenotypic maintenance of different cell types [8]. Several techniques, including physical, chemical, and biological agents, are commonly used to remove cells from different tissues [9,10]. The collagen content, as a major component of the ECM, is used as a measure of the ECM damage during the decellularization process [11]. Decellularization methods remove most of the immunogenic components and it has been described that decellularized ECM materials elicits an anti-inflammatory immune response, reducing the risk of implant rejection [12,13,14]. In this sense, the use of this kind of materials for preparing three-dimensional scaffolds allows the natural cellular environment to be mimicked and promotes specific cellular responses necessary for tissue regeneration [15,16]. The possible employment of decellularized ECM material for regenerative medicine, requires the evaluation of the in vitro response of macrophages after exposure with this material due to the key role of these cells in both the host response to biomaterials and the inflammation/healing processes. These cells present a high functional plasticity that allows them to polarize into proinflammatory (M1) and anti-inflammatory (M2) phenotypes characterized by different cell surface markers and cytokines [17]. M1 macrophages are characterized by tumor necrosis factor-alpha (TNF-α) release and high expression of CD80 and inducible nitric oxide synthase (iNOS) [18]. M1 cells also produce reactive oxygen species (ROS) that can damage neighboring cells and promote the proinflammatory response [19]. On the other hand, the M2 phenotype is involved in tissue repair by the secretion of anti-inflammatory cytokines, such as interleukin-10 (IL-10) and tumor growth factor-β (TGF-β), that inhibit proinflammatory cytokines released by M1 macrophages [20]. M2 macrophages are characterized by the expression of surface markers, such as scavenger receptor (CD163), mannose receptor (CD206), and MHC class II receptors. M2 macrophages can be further divided into the subcategories M2a, M2b, M2c, and M2d. M2a (wound-healing macrophages) play an important role in parasite killing, allergy, and fibrosis progression, and are characterized by the cell surface expression of CD206 mannose receptors. M2b macrophages regulate the immune response and are characterized by the cell surface expression of CD86 receptors [21]. M2c macrophages exert anti-inflammatory and profibrotic functions, such as ECM remodeling, and are characterized by cell surface expression of scavenger receptors for the hemoglobin-haptoglobin complex CD163 [22,23]. The M2d phenotype has only been described in mice and is characterized by increased production of IL-10 and vascular endothelial growth factor (VEGF) and low expression of TNF-α and IL-12 [24]. On the other hand, macrophage phagocytosis is considered as the main sign of the macrophage function due to its significance in the uptake and degradation of both the infectious agents and senescent cells. Thus, this process is of key importance not only for the immune response and inflammation but also in tissue remodeling [25].

In this context, the effects of ECM bio-scaffold materials on the macrophage function as well as on the balance between the M1-mediated inflammation and M2-mediated regeneration must be evaluated because the ratio of regulatory/proinflammatory macrophages has been shown to be a predictor of favorable outcomes in multiple studies [26,27]. Furthermore, it is necessary also to analyze the macrophage immunocompetence against pathogenic microorganisms after contact with decellularized ECM materials to assess the functional role of these cells in a possible infection scenario.

In the present work, we studied the macrophage polarization and immunocompetence against *Candida albicans* (an opportunistic fungus that easily colonizes host tissues) after their exposure to four decellularized adipose matrices (DAMs) obtained by two different protocols that process AT harvested from human and porcine species.

## 2. Results and Discussion

Modulation of the macrophage phenotype exposed to ECM-based biomaterials can improve the tissue remodeling process after implantation of these bio-scaffolds. The most important factors involved in the specific response of macrophages to these biomaterials are their tissue source and the efficiency of the decellularization method. Here, we provide a comprehensive analysis of the macrophage M1/M2 polarization and their immunocompetence after the exposure to coatings of DAMs obtained by two different decellularization protocols that process AT harvested from human and porcine species. In this sense, and along this work, the decellularization efficiency and native ECM protein conservation were related with the observed macrophage response.

### 2.1. Characterization of Decellularized Extracellular Matrices

The goal of the decellularization processes is the effective removal of cellular and nuclear components while preserving the composition and structure properties of the native ECM proteins [28]. Currently, there is no universally accepted standardized protocol for the decellularization of AT. In general, the methods include a combination of physical (freezing, pressure, sonication, agitation) and chemical (enzyme, detergent, acid, alkaline) treatments of the tissue. However, it is accepted by the research community that each decellularization treatment affects the biochemical composition of the remaining ECM materials, which, in turn, carries functional and pathophysiological implications to its regenerative capacities [29]. Thus, AT decellularization must effectively remove cellular components and lipids, minimizing the loss of native ECM components [30,31]. In this study, two decellularization protocols were used to produce human and porcine DAMs: Method 1 based on fine-tuned enzyme incubations and method 2 based on organic solvents. Specifically, for AT, an effective delipidation is also mandatory. Thus, specific decellularization criteria were employed, quantifying not only the remnant DNA but also the adipose lipids after enzymatic and organic solvent treatment in the above-mentioned DAMs.

#### 2.1.1. Quantification of the Remnant DNA

All the DAMs (hDAM1, hDAM2, pDAM1, and pDAM2) meet the decellularization criteria established by the research community of less than 50 ng DNA/mg dry weight (Table 1). Results showed statistical differences (*** *p* < 005) between methods, only in porcine DAMs (pDAM1 vs. pDAM2). Specifically, pDAM2 showed statistically higher remnant DNA (24.8 ± 2.05 ng/mg) in comparison with the other studied DAMs (*** *p* < 005). It is important to note that there were also significant differences (*** *p* < 005) after comparing DAMs from different sources, i.e., human and porcine AT, obtained by the same method (hDAM1 vs. pDAM1 and hDAM2 vs. pDAM2).

#### 2.1.2. Quantification of the Remnant Lipids by Liquid Chromatography

Besides the remnant DNA, in AT decellularization the delipidation of the tissue is also a critical parameter. So, remnant lipids quantification was performed for all assayed DAMs. The results in Table 2 show the efficiency of the methods to remove lipids from human and porcine AT. However, differences between methods are evidenced; in this sense, the enzymatic method was clearly more effective for delipidation of DAMs. Results showed 0.1 ± 0.0% (*w/w*) remnant lipids (0.8 ± 0.1 µg/mg) for human (hDAM1) and 0.3 ± 0.1% (*w/w*) remnant lipids (3.0 ± 1.0 µg/mg) for porcine (pDAM1). DAMs obtained after the organic solvent method (2) showed a maximum of 7.0 ± 0.2% (*w/w*) remnant total lipids (69.6 ± 2.1 µg/mg) in porcine (pDAM2), and fewer for human (hDAM2) with 0.6 ± 0.2% (*w/w*) (5.8 ± 1.5 µg/mg). Concerning the types of lipids analyzed, hDAM2 and pDAM2, obtained from AT decellularized by treatment with organic solvent (isopropanol) showed a higher content of triacylglycerols than hDAM1 and pDAM1, obtained by enzymatic treatment with lipoprotein lipase and benzonase. These results evidenced the specific action of lipoprotein lipase on triacylglycerols contained in adipocytes, leading to low levels of this kind of lipid in hDAM1 and pDAM1. In addition, pDAM2 showed much higher values of triacylglycerols than hDAM2, probably due to the higher fat content in porcine AT than in human AT.

#### 2.1.3. Mass Spectroscopy Characterization

The ECM proteins conserved in the four DAMs were evaluated through LC-MS/MS in a comparative analysis considering native and decellularized AT. The native human and porcine AT proteomic analysis resulted in a reading of 1459 and 550 peptides, respectively. UniProtKB-SwissProt database and protein atlas (www.proteinatlas.org (accessed on 4 January 2021)) allowed us to identify 52 human and 38 porcine ECM-specific proteins, including 14 and 11 basement membrane (BM)-specific peptides from human and porcine origin, respectively. First expressed in early embryogenesis, BM proteins are self-assembled on competent cell surfaces through binding interactions among laminins, type IV collagens, nidogens, and proteoglycans. The fact that BMs are a substrate on which cells attach, grow, and differentiate underlines their biological importance [32].

**Figure 1:**
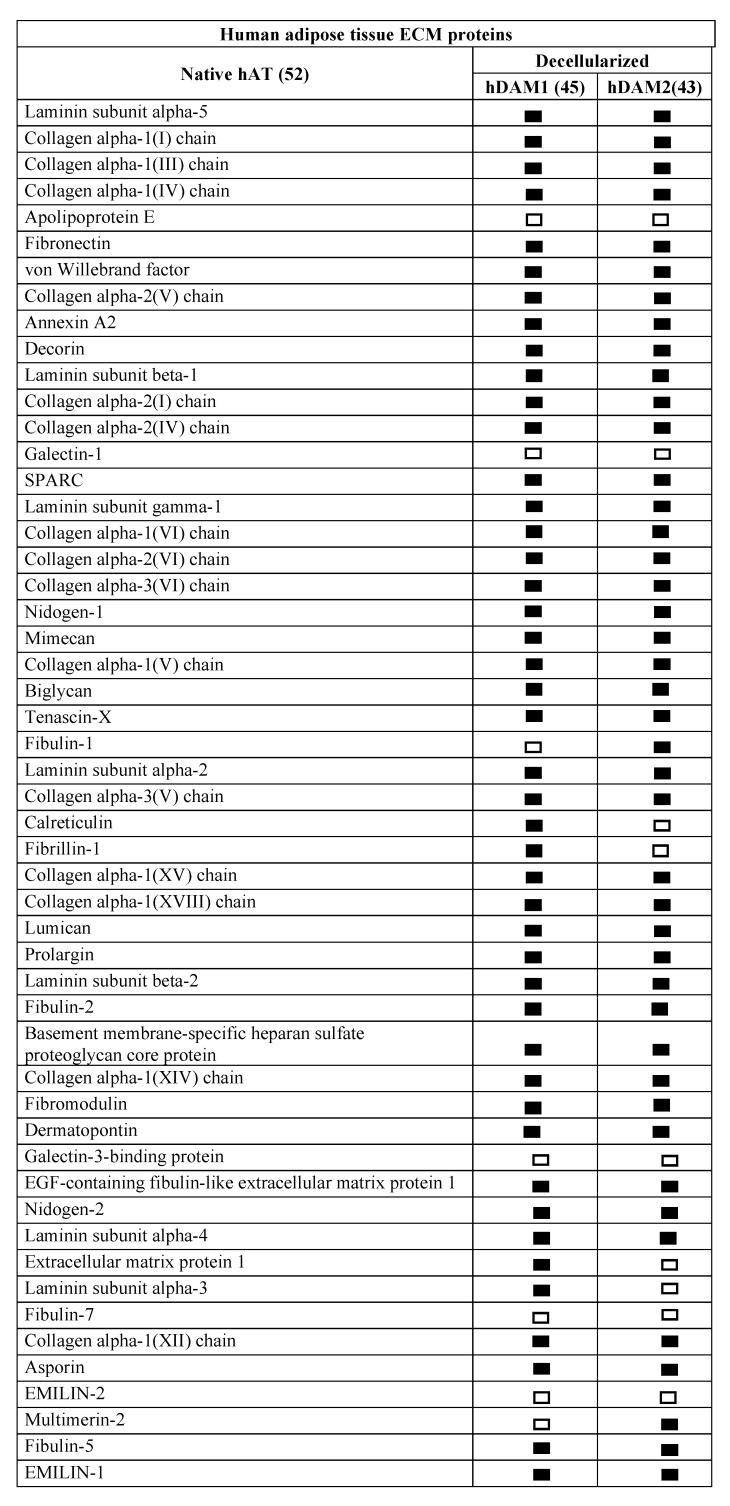
Human extracellular matrix (ECM) protein composition before (native) and after adipose tissue (AT) decellularization by method 1 (hDAM1) and method 2 (hDAM2).

**Figure 2:**
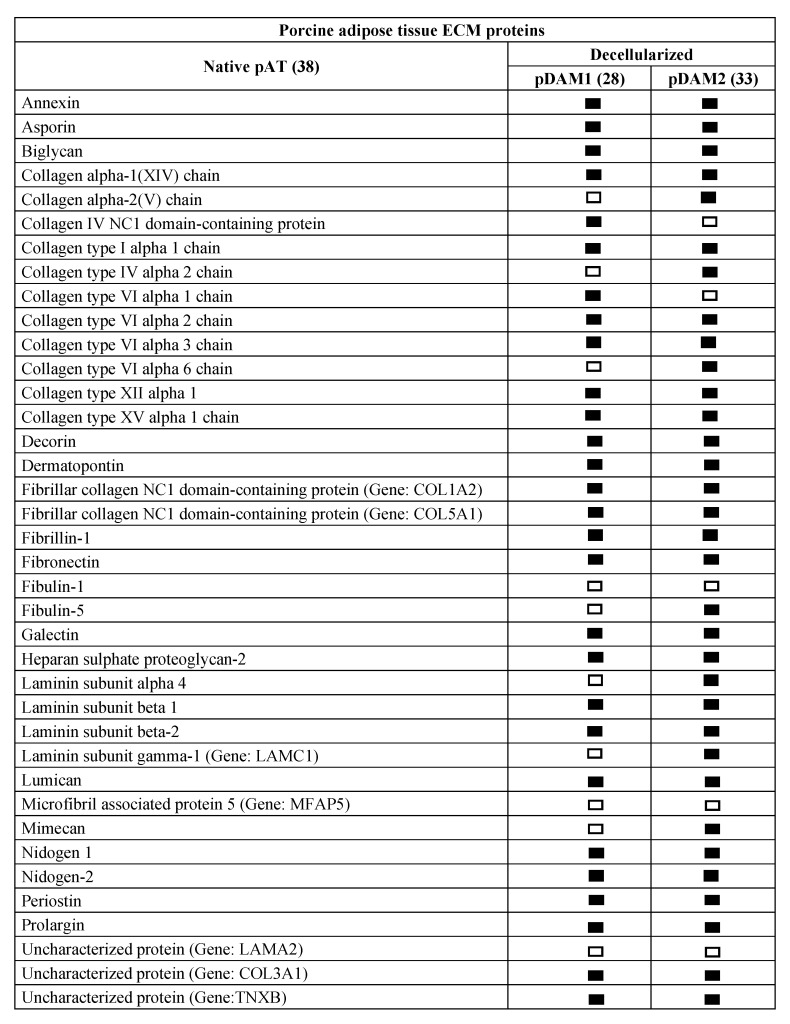
Porcine ECM protein composition before (native) and after AT decellularization by method 1 (pDAM1) and method 2 (pDAM2).

Comparative proteomic analysis of ECM proteins from native AT and DAMs was done for human and porcine samples. Human proteomic results are shown in Figure 1 and Supplementary Materials Table S1 and those from porcine origin in Figure 2 and Supplementary Materials Table S2. Related to human, the enzymatic method (hDAM1) conserved 86.5% (45 proteins) of native ECM proteins while the organic solvent method (hDAM2) conserved 82.7% (43 proteins). Both DAMs conserved all the native BM proteins except laminin subunit α3, which is not identified in hDAM2. Concerning the porcine AT, pDAM1 conserved 73.7% (28 proteins) of native ECM proteins while pDAM2 conserved 86.8% (33 proteins). All the native BM proteins except collagen type IV NC1 domain-containing protein and protein coding gene LAMA2 were conserved in pDAM2. On the other hand, pDAM1 conserved all BM proteins but collagen type IV α2 chain protein and 3 laminins (laminin subunit α4 and laminin’s coding gene LAMA2 and LAMC1). As it is well known, collagen type IV is the major structural component of the BM, and it consists of a family of six homologous (IV) chains, designated α1–α6. Each chain is characterized by a long collagenous domain of approximately 1400 residues of Gly-X-Y repeats, interrupted by approximately 20 short noncollagenous sequences, and by a noncollagenous (NC1) domain of 230 residues at the carboxyl terminus. These proteomic studies show that more than 80% of native proteins are conserved in human-derived DAMs and more than 70% in porcine-derived ones. In addition, this approach evidenced that some proteins are differentially preserved depending on both the AT origin and the decellularization method employed; this issue will be deeply discussed below in Section 2.4.

Supplemental Table S1. Human AT ECM proteins identified by mass spectrometry before (hAT) and after decellularization by method 1 (hDAM1) and method 2 (hDAM2). 

Supplemental Table S2. Porcine AT ECM proteins identified by mass spectrometry before (pAT) and after decellularization by method 1 (pDAM1) and method 2 (pDAM2).

### 2.2. Coated Total Protein

All the DAMs successfully coated the 6-well polystyrene plates and glass coverslips. Table 3 showed the total protein coated for each type of DAM. Results showed a range of 0.9–1.5 µg protein/cm^2^ coated on the polystyrene plates and 1.1–1.4 µg protein/cm^2^ on the coverslips. These results agree with previously reported extracellular matrix coatings for cell culture [32,33] and demonstrate that a successful coating is possible with the four DAMs on very useful substrates for the in vitro studies carried out in this work.

### 2.3. Effects of DAMs on the Polarization of RAW-264.7 Macrophages towards M1 and M2 Phenotypes 

Implantation of a biomaterial induces a host reaction that determines the outcome of the integration and the biological performance of the implant. In this context, macrophages play an essential role due to their functional plasticity, which allows them to respond to their local environment, changing their phenotype [34,35]. During the early stages of the inflammatory process, M1 macrophages are potent effector cells with proinflammatory behavior. However, at later stages, M2 macrophages reduce these inflammatory and adaptive Th1 responses by producing anti-inflammatory cytokines promoting angiogenesis, tissue remodeling, and repair [36]. This process, called “macrophage polarization”, has been described during wound repair in a diverse array of tissues, including heart, lung, muscle, skin, and bone [37,38,39]. 

**Scheme 1:**
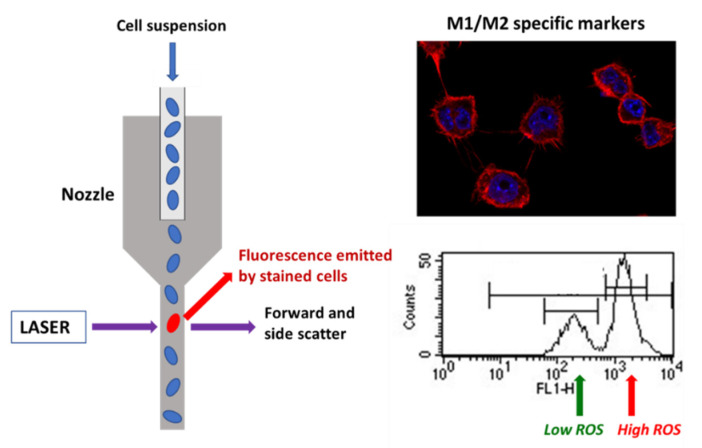
Evaluation of different specific markers of M1 and M2 macrophage phenotypes by flow cytometry and confocal microscopy after contact of RAW-264.7 macrophages with DAMs.

The goal of tissue decellularization is thorough removal of cells and cell components while retaining the composition of the native ECM to the extent possible. Decellularized ECM with nucleic materials incompletely removed or degraded could induce a host innate immune response and modulate the phenotype of the cells involved in tissue remodeling. Thus, some studies and a recently published standard guide suggest that decellularized ECM materials should contain ≤50 ng DNA/mg ECM dry weight [40,41]. When tissues have been efficiently decellularized, the ECM-specific structural and functional components that remain (collagen, laminin, fibronectin, growth factors, etc.) are relatively well conserved across mammalian species and, therefore, should not evoke adverse immune reaction [42]. Among all biological matrices, decellularized extracellular matrix has recently emerged as a promising biomaterial that is being used alone or in combination with other biomaterials in the fields of tissue engineering and regenerative medicine, both preclinically and clinically. Decellularized extracellular matrix provides a native cellular environment that combines its unique composition and architecture. It can be widely obtained from native organs of different species after decellularization and provides the necessary signals for cell guidance [42]. In the present study, the DNA content quantified after both decellularization methods was lower than 5 ng/mg for hDAM1, hDAM2, and DAM1 and 24.8 ng/mg for pDAM2 DNA/mg ECM dry weight, thus indicating the high efficiency of these processes and the possible absence of adverse immune reaction induction. Some authors have indicated that decellularized ECM can promote a switch from a M1-like macrophage population post-implantation to a population enriched in M2-like macrophages [27]. In this context, we analyzed in the present work the effects on macrophage polarization induced by the four DAMs obtained from porcine and human AT by the enzymatic method 1 (pDAM1, hDAM1) and organic solvent method 2 (pDAM2 and hDAM2). For this purpose, specific M1 and M2 markers as well as intracellular oxygen species (ROS) content were measured by flow cytometry and confocal microscopy as shown in Scheme 1. In addition, the secretion of the proinflammatory cytokine TNF-α was analyzed by ELISA methods.

**Figure 3:**
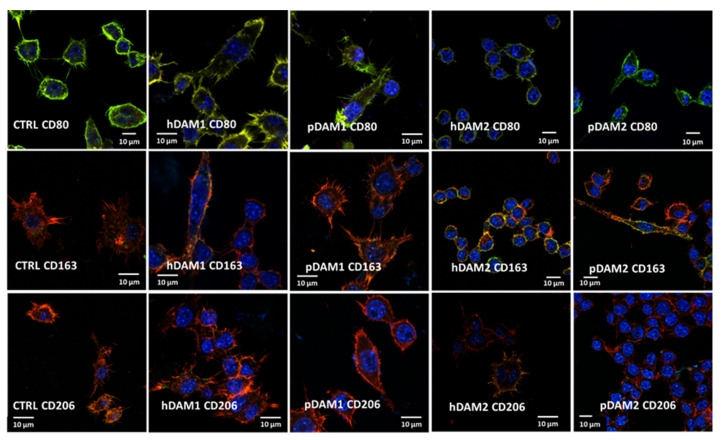
M1 and M2 specific markers observed by confocal microscopy in RAW-264.7 macrophages after 24 h of culture on control surface (CTRL) and human or porcine DAMs obtained by the enzymatic method (hDAM1and pDAM1) and organic solvents treatment (hDAM2 and pDAM2). Top panel: CD80 in red, cytoskeleton actin filaments in green (colocalization results in yellow coloration) and nuclei in blue. Middle and lower panels: CD163 and CD206 are observed in green, cytoskeleton actin filaments in red (colocalization results in yellow coloration), and nuclei in blue.

The expression of specific markers of the M1 proinflammatory phenotype (CD80) and M2 reparative phenotype (CD163 and CD206) was observed by confocal microscopy and quantified by flow cytometry. Figure 3 evidences the expression of all these markers in RAW-264.7 macrophages cultured on control surfaces and on the different DAMs. Confocal images allow us to observe the characteristic morphology of macrophages on these materials as well the homogenous distribution of all the studied markers on the cell surface. In addition, the staining of the actin filaments reveals the correct cytoskeleton assembly on spreading macrophages on all these surfaces. In summary, these images reflect the correct cell adhesion to the different DAMs when compared to control surfaces, and the high viability of all the cultures. 

**Figure 4:**
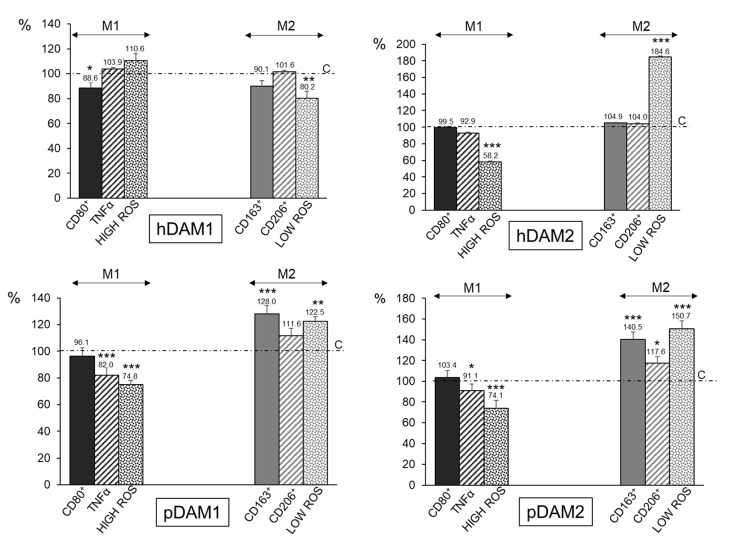
Percentage of proinflammatory M1 RAW-264.7 macrophages (characterized by CD80^+^, TNF-α, and HIGH ROS) and reparative M2 macrophages (characterized by CD163^+^, CD206^+^, and LOW ROS) after 24 h of culture on human (h) and porcine (p) DAMs obtained by either enzymatic methods (hDAM1 and pDAM1) or treatment with organic solvents (hDAM2 and pDAM2). All the values are shown as percentages referred to the corresponding control value of each parameter taken as 100%. Statistical significance: *** *p* < 0.005, ** *p* < 0.01, * *p* < 0.05 (comparison of the results obtained in the presence of matrix with their respective controls in the absence of matrix).

In order to quantify the percentage of M1 and M2 phenotypes, flow cytometry analyses were performed with macrophages after 24 h of exposure to the different DAMs. Figure 4 shows together M1 and M2 biological parameters of macrophages after DAMs contact. All the values are shown as percentages referred to the corresponding control value of each parameter taken as 100%. With respect to the proinflammatory phenotype M1, the expression of the CD80 surface receptor (CD80^+^ cells), as a prototypical M1 marker, the release of the proinflammatory cytokine TNF-α, and the percentage of the cell population with high intracellular oxygen species (ROS) content were evaluated [43]. The results highlight that none of the four DAMs studied induce the polarization of the macrophages towards the proinflammatory M1 phenotype. In fact, the expression of the CD80 marker is significantly lower after hDAM1 exposure, and it does not change after contact with hDAM2, pDAM1, and pDAM2. Moreover, there is a significant decrease in the levels of TNF-α released by macrophages after culture on pDAM1 and pDAM2. In the same way, a significant decrease in the percentage of cell population with a high ROS content was also observed when macrophages were cultured on hDAM2, pDAM1, and pDAM2. These results suggest a protective role of these DAMs against oxidative stress, in agreement with other authors [44]. 

To identify the macrophage population with the M2 phenotype, the expression of the CD163 (hemoglobin-haptoglobin scavenger receptor) and CD206 (mannose receptor) as typical markers of M2c and M2a macrophages, respectively, together with the percentage of the cell population with low intracellular ROS content were studied (Figure 4). The results obtained with pDAM1 and pDAM2 indicate a significant increase of the percentage of CD163^+^ macrophages together with a significant increase in the percentage of the cell population with low ROS content. Similarly, when macrophages were cultured on pDAM2, a significant increase in the percentage CD206^+^ cells was observed. As previously mentioned, M2c macrophages express high levels of the surface marker CD163, and they have been shown to infiltrate wound sites during early phases (1–2 days) of wound healing in humans [45,46]. Other authors demonstrated that M2c macrophages promoted angiogenesis in vitro and in vivo [47,48]. In the present study, a significant increase of CD163^+^ macrophage population was observed after their culture on both porcine DAMs, independently of the decellularization method employed. Considering the important role of the CD163^+^ macrophages in the wound healing process, our results highlight that these pDAMs could be proposed as excellent candidates to produce three-dimensional materials for tissue regeneration. Additionally, our results agree with those obtained by Rui-Xin Wu et al., in which it was found that bone ECM hydrogels were more likely to polarize macrophages towards the M2 phenotype, leading to enhanced tissue regeneration [49]. Finally, LoPresti et al. recently studied the macrophage response to porcine small intestine ECM biomaterials decellularized with nitric oxide, hydroxyl radical, or both, in comparison to a standard method using peracetic acid. Specifically, they hypothesized that decellularization of ECM with radical oxygen or nitrogen species would promote enhanced anti-inflammatory and antioxidant macrophage polarization, which would improve healing after muscle injury [50]. In summary, as it is shown in Table 4, the DAMs from porcine origin favor the macrophage M2 phenotype, independently of the decellularization method employed. In contrast, macrophages cultured on human DAMs do not show significant changes. 

### 2.4. Effects of Decellularized Extracellular Matrices on the Immunocompetence of RAW-264.7 Macrophages Against Candida albicans

#### Phagocytosis of *Candida albicans* by RAW-264.7 Macrophages

**Figure 5:**
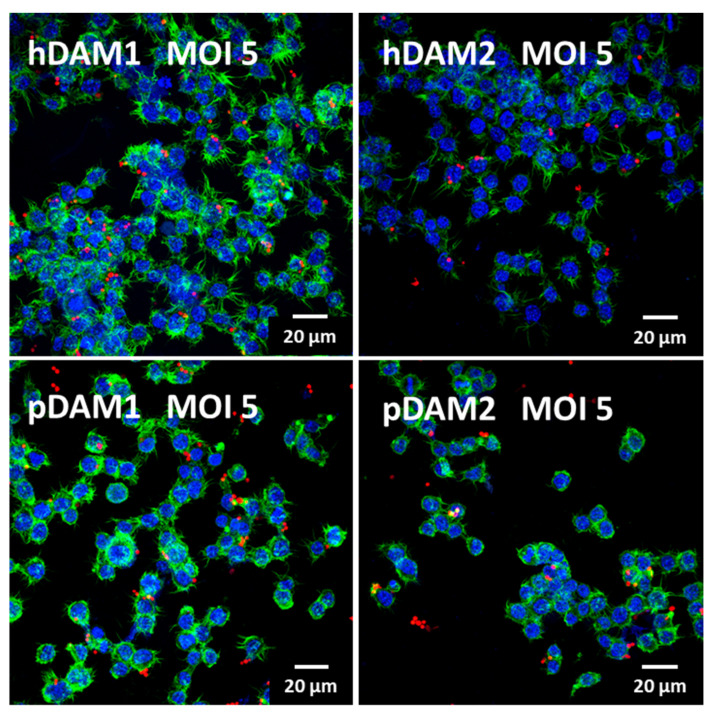
Confocal microscopy images of RFP-*C. albicans* (in red) ingested by RAW macrophages at MOI 5 after culture on human (h) and porcine (p) DAMs obtained by either enzymatic methods (hDAM1 and pDAM1) or treatment with organic solvents (hDAM2 and pDAM2). Nuclei were labeled in blue with 4′,6-diamidino-2-phenylindole (DAPI), and actin cytoskeleton were labeled in green with FITC-phalloidin.

Macrophages are phagocytic cells that are actively recruited to eliminate foreign particles and debris arising from tissue homeostasis or damage. Phagocytosis is an evolutionarily conserved mechanism that plays an essential role in the defense against disease, by which immune cells take up microbial pathogens and tumor cells for degradation. Phagocytes, such as macrophages and neutrophils, are the primary line of defense in infections by extracellular pathogens, such as fungi and bacteria. *Candida albicans* is a commensal fungus that in certain conditions behaves like a true pathogen, causing different infections (oral cavity, gastrointestinal and urogenital tracts) or systemic disorders with high mortality rates among immunocompromised populations [51,52]. Therefore, considering the fundamental role of macrophages in the resolution of bacterial/fungus infection, the assessment of the effects of these DAMs on the immunocompetence of macrophages against a pathogen (like *C. albicans*) is essential to determine whether they could be used as biomaterials for tissue regeneration. In this sense, a fungal phagocytosis assay was performed to evaluate the functional role of macrophages after their exposure to the four DAMs obtained in this study. *Candida albicans* phagocytosis by macrophages cultured on human (h) and porcine (p) DAMs was observed by confocal microscopy and quantified by flow cytometry. Figure 5 shows representative confocal microscopy images of *C. albicans* ingested by RAW macrophages after exposure to human and porcine DAMs obtained by either enzymatic methods or by organic solvents treatment. As it is shown, in these experimental conditions, macrophages properly adhered to the different DAMs, maintaining intact actin filopodia as well as their phagocytosis capability. With the aim of quantifying fungal phagocytosis after exposure to the four DAMs and in order to evaluate possible differences among them, a thorough flow cytometry analysis was performed. This kind of analysis has been recently developed by our group as a confident and very useful method to explore macrophage immunocompetence after exposure to different biomaterials [53]. 

**Figure 6:**
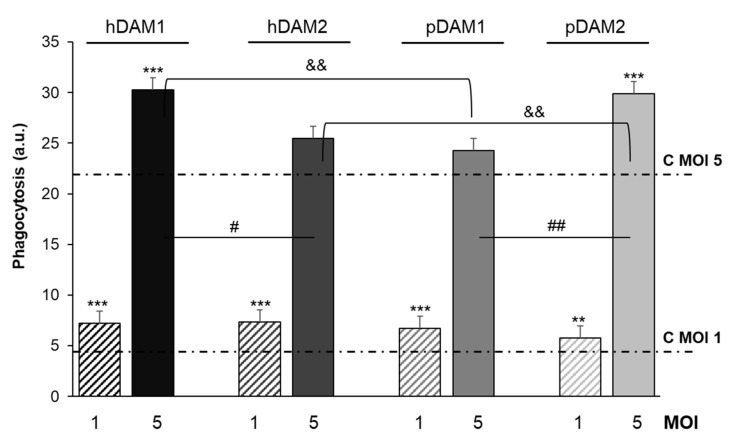
RFP-*Candida albicans* phagocytosis by RAW-264.7 macrophages cultured on human (h) and porcine (p) DAMs obtained by either enzymatic method (hDAM1 and pDAM1) or treatment with organic solvents (hDAM2 and pDAM2), after 45 min of treatment with the fungus (MOI 1 and MOI 5) and flow cytometric analysis. Statistical significance: *** *p* < 0.005, ** *p* < 0.01 (comparison of the results obtained in the presence of matrix at MOI 1 and MOI 5 with their respective controls without matrix, lines C MOI 1 and C MOI 5). ^#^ *p* < 0.05, ^##^ *p* < 0.01 (hDAM1 vs. hDAM2, pDAM1 vs. pDAM2, at MOI 5). ^&&^ *p* < 0.01 (hDAM1 vs. pDAM1 and hDAM2 vs. pDAM2, at MOI 5).

*Candida albicans* phagocytosis by macrophages cultured on these DAMs was quantified by flow cytometry. Figure 6 shows fungal phagocytosis at two different ratios (MOI 1 and 5), by macrophages after exposure to human (h) and porcine (p) DAMs obtained by either enzymatic method (hDAM1 and pDAM1) or treatment with organic solvents (hDAM2 and pDAM2). The previous exposure of macrophages to these DAMs increased their fungal phagocytosis independently of the MOI employed. In addition, significant differences were obtained in the conditions studied, evidencing the sensitivity of theses assays. 

The results highlight that at a lower macrophage-fungus ratio (MOI 1), the macrophages previously cultured on all these DAMs present a significant higher phagocytic capacity compared to that shown by control macrophages (C MOI 1). On the other hand, at a higher ratio (MOI 5), macrophages exposed to the four DAMs exhibited higher fungal phagocytosis compared to control macrophages (C MOI 5), although this increase was significant only with hDAM1 and pDAM2. Moreover, it should also be noted that, at this ratio, these differences could be attributed to the DAMs origin (human or porcine) as well as to the decellularization method by which they were obtained. In fact, after comparing the human DAMs, hDAM1 vs. hDAM2, we observed a significant increase in fungal phagocytosis after exposure to hDAM1 (enzymatic method). However, for porcine DAMs, the phagocytic capacity of macrophages was significantly higher on pDAM2 (organic solvents treatment) compared to pDAM1. Finally, after comparing DAMs of different origin (human and porcine) and obtained by the same decellularization method, a significant higher fungal phagocytosis of macrophages cultured on hDAM1 than on pDAM1 and on pDAM2 than on hDAM2 was observed. It is generally accepted that the higher presence of the fungus increases the macrophage sensitivity to its environment. The phagocytosis results at MOI 5 highlight the sensitivity of this assay to detect differences in the macrophage response to these four DAMs. Recently, regulation of macrophages by extracellular matrix composition has been shown [54]. In the present study, fungal phagocytosis results are related to the biochemical composition (DNA and lipid content) of the four DAMs. Moreover, the differentially preserved proteins and their relationship to the phagocytosis capability of macrophages are also shown. A deep discussion regarding the key role of these proteins is included below, to justify properly the obtained phagocytosis results. 

**Figure 7:**
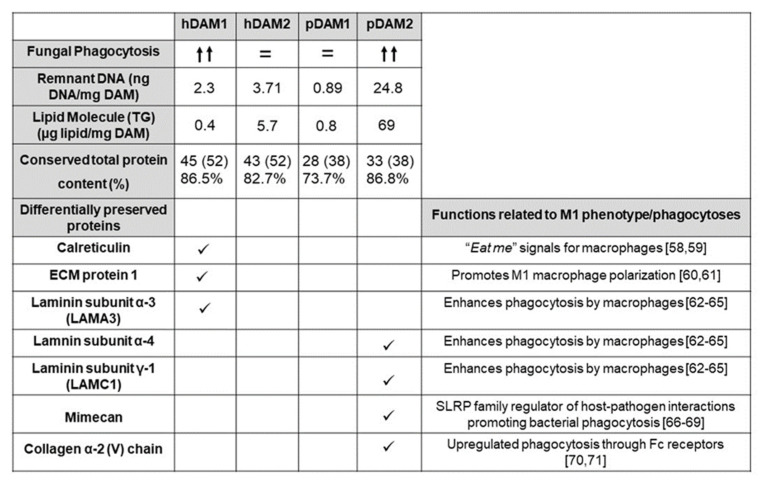
Fungal phagocytosis by macrophages exposed to hDAM1, hDAM2, pDAM1, and pDAM2, related to the content of DNA, lipids, and proteins of these matrices and to their differentially preserved proteins.

The ECM not only acts as structural support for cells within tissues but also plays an important signaling role through interactions with adhesion receptors and growth factors, among other molecules [55]. During homeostatic maintenance and in response to injury, the ECM is subject to extensive and continuous remodeling and all these functions depend on its biochemical properties. In this context, the total protein content and the differentially preserved proteins of the four DAMs evaluated in the present study are shown in Figure 7. As mentioned in the section corresponding to the characterization of the DAMs by proteomic analysis, after the decellularization process of human and porcine AT by enzymes (1) and organic solvents (2), we found differences in some ECM proteins. Specifically, calreticulin, ECM protein 1, and laminin subunit α3 (LAMA 3) are conserved only in hDAM1 and not in hDAM2. On the other hand, we found that collagen type V, fibulin 5, laminin α4, laminin γ1, and mimecan are preserved in pDAM2 and not in pDAM1. Interestingly, in the present work, we observed higher fungal phagocytosis (MOI 5) by macrophages exposed to hDAM1 and pDAM2, which present a higher total protein content. Moreover, specific proteins were differentially preserved in these two DAMs (Figure 7). These results agree with several studies that evidence the influence of the ECM proteins on the immune cell function [56]. Furthermore, recent studies have also highlighted that the ECM composition influences the adhesion of macrophages and their phagocytosis capability [54,57]. Protein calreticulin (CRT) is known to be one of the “eat me” signals for macrophages [58,59]. In our study, the presence of CRT in hDAM1 could promote fungal phagocytosis by macrophages, thus explaining their greater phagocytic capacity observed after exposure to this DAM. Another preserved protein in hDAM1 is ECM1, a multifunctional protein that interacts with most other ECM proteins and has an important function in promoting M1 macrophage polarization, which is critical for controlling inflammation and tissue repair [60,61]. Specifically, certain ECM proteins, such as fibronectin and laminin, have been shown to increase the phagocytic activity of macrophages [62]. Moreover, recent research highlights the prominent role of laminin proteins on the regulation of different immune cell types, including macrophages [63]. In our study, several laminins are preserved in hDAM1 and pDAM2. Bohnsack et al. demonstrated that the interaction of laminin with human macrophages enhanced their phagocytic function, most likely through a receptor Fc-mediated mechanism [64]. Leslie et al. demonstrated a strong causal relationship between macrophage activation and laminin adhesion [65]. The mimecan protein, also named osteoglycin (OGN), preserved only in pDAM2, belongs to the small leucine-rich proteoglycan (SLRP) family of proteins, which are well known for their well-timed action on shaping the architecture and organization of collagen-rich extracellular matrices in certain organs [66]. The structure and function of OGN is still not clear [67]; however, OGN has been found in both neutrophils and monocytes/macrophages [68]. Recent studies have demonstrated that lumican (Lum), another member of the SLRP family, is emerging as a regulator of host–pathogen interactions promoting bacterial phagocytosis [69]. Therefore, probably mimecan protein, present in pDAM2, might promote the *Candida albicans* phagocytosis by macrophages, as has been described with respect to the relationship between lumican in the *Pseudomonas aeruginosa* phagocytosis. Finally, regarding the preserved collagen in pDAM2, it is known that this protein plays a complex multifaceted role in the regulation of immune cells [70]. More specifically, it has been shown that the adhesion to collagen generally reduces the inflammatory response of macrophages and is thought to have a critical regulatory role for innate immune responses. In fact, Kirkham et al. demonstrated that the interaction of macrophages with ECM protein collagen upregulated phagocytosis through Fc receptors [71]. All these arguments highlight the close relationship between the ECM biochemical composition and the macrophage’s functional role.

## 3. Materials and Methods

### 3.1. Human and Porcine Adipose Tissue Decellularization

Porcine AT was harvested from a local food company (JAUCHA S.L., Navarra, Spain) and human AT from Biopredict International (Saint Grégoire, France) according to the French Ministry of Higher Education and Research permission AC-2013-1754. 

Human AT was cleaned of blood using a scalpel and serial cleaning with a high quantity of ultra-pure water. Additionally, tissue was cleaned with ACK Lysis buffer (Sigma-Aldrich Corporation, St. Louis, MO, USA) according to the manufacturer′s instructions, obtaining cleaned tissue free of blood components. Porcine AT was supplied cleaned, free of blood components. Both human and porcine tissues were creamed using a beater and stored at −20 °C in 400-mL containers until the decellularization process. 

For tissue decellularization, previously creamed porcine and human tissues were maintained at room temperature for defrosting; afterwards, the tissues were homogenized on ice using a Polytron (PT3100) with two different rods at 12,000 rpm for 5 min. Homogenized tissues were centrifugated at 5000 rpm for 5 min with ultrapure water to produce the phase separation of lipids, which were discarded, manually conserving the protein pellet accumulated during centrifugation at the bottom of the container. The pellets were conserved at −20 °C or treated to finish the decellularization protocol. In this study, two different treatments were used for ongoing processing of the tissues. 

The first protocol was based on TECNALIA′s own enzymatic protocol [72]. Briefly, the protein pellets were treated with l50 u./100 mg of lipoprotein lipase (Merck Life Science SL, Madrid, Spain) in phosphate buffer saline (PBS, Merck Life Science SL, Madrid, Spain) within 0.5% (*v/v*) triton^®^X-100 (Merck Life Science SL, Madrid, Spain) for 48 h at 37 °C and continuous orbital shaking. Afterwards, centrifugations at 900× *g* for 5 min were carried out to wash the samples 8 times with PBS supplemented with 1% (*v/v*) antibiotic-antimycotic solution (Gibco, BRL, United Kingdom) and a last wash with Ultrapure milli-Q water. The materials obtained were frozen and lyophilized for drying. The second enzymatic treatment was carried out with 1500 u./mg of Benzonase^®^ (Emprove^®^-bio, Merck Life Science SL, Madrid, Spain) in PBS and 2 mM magnesium chloride (Merck Life Science SL, Madrid, Spain) for 72 h at 37 °C and continuous orbital shaking. Afterwards, the material was cleaned toughly with PBS and antibiotic-antimycotic solution (Gibco, BRL, United Kingdom) and with ultrapure water for lyophilization until completely dry. The decellularized adipose matrices (DAMs) obtained by this enzymatic method were identified as porcine and human DAM1 (pDAM1 and hDAM1, respectively). 

For the second protocol of tissue decellularization, pellets were treated with pure isopropanol (Merck Life Science SL, Madrid, Spain) overnight under orbital shaking at room temperature. Afterwards, the material was thoroughly cleaned as previously described and treated with 1% (*v/v*) triton x-100 and 0.1% (*v/v*) ammonium hydroxide (Merck Life Science SL, Spain) for 44 h. Then, the material was thoroughly cleaned as previously described and lyophilized until completely dry. The DAMs obtained by this organic solvent method were identified as porcine and human DAM2 (pDAM2 and hDAM2, respectively). 

In order to create a fine-grained powder suitable for processing, all the lyophilized materials (pDAM1, hDAM1, pDAM2, and hDAM2) were milled using a mixer mill (Retsch MM400) preceded by freezing in liquid nitrogen and conserved at 4 °C in a vacuum desiccator.

### 3.2. Quantification of the Remnant DNA

Remnant single- and double-strain DNA were analyzed by quantitative real-time polymerase chain reaction (qRT-PCR). The DNA was extracted from the DAMs by the QiAmp kit (Qiagen, Madrid, Spain) according to the manufacturer′s instructions. For the absolute quantification, a standard curve of known-concentration DNA samples was obtained. For human samples, DNA blood was extracted by the Qiamp DNA Blood MiniKit (Qiagen, Madrid, Spain) according to the manufacturer’s instructions and for porcine PIG1 DNA was used. Solutions containing 50 ng/mL DNA were obtained and analyzed by an ND-1000 spectrophotometer (NanoDrop Technologies, Wilmington, Delaware, USA) and serial dilutions were prepared. Detection was carried out by the specific sequence of the human and porcine gen hemoglobin, beta (HBB, 11p.15.5 and 9p.2.4, respectively) and analyzed using a LightCycler 480II (Roche Diagnostics, Madrid, Spain).

Reactions were performed in triplicate; data are shown as remnant DNA (ng) for dry weight DAM (mg). The detection limit of the technique was estimated in approximately 20 pg of DNA. The DNA quantification was carried out by DNAdata, a Genetic Disease Diagnostic Centre authorized by The Basque Country Government.

### 3.3. Mass Spectroscopy Quantification of the Remnant Lipids 

Quantitative analysis of total lipid content in DAMs was carried out by liquid chromatography coupled with Q-TOF mass spectrometry (UPLC/Q-TOF MS): An ACQUITY UPLC system from Waters (Milford, MA, USA), equipped with a binary solvent delivery pump, an auto-sampler, and a column oven. The extracts were injected onto a Waters column (Acquity UPLC HSS T3 1.8 μm, 100 mm × 2.1 mm), which was heated to 65 °C. Mobile phases consisted of acetonitrile and water with 10 mM ammonium acetate (40:60, *v/v*) (phase A) and acetonitrile and isopropanol with 10 mM ammonium acetate (10:90, *v/v*) (phase B). Separation was carried out in 13 min under the following conditions: 0–10 min, linear gradient from 40% to 100% B; 10–11 min, 100% B; and finally, equilibration of the system with 40% B (*v/v*) for 2 min prior to the next injection. The flow rate was 0.5 mL/min and injection volume were 5 μL. All samples were kept at 4 °C during the analysis. All UHPLC-MSE data were acquired on a SYNAPT G2 HDMS, with a quadrupole time of flight (Q-ToF) configuration, and (Waters) equipped with an electrospray ionization (ESI) source that can be operated in both positive and negative modes. The mass spectrometry analysis was performed in the Lipidomic Core Facility-SGIKER at the University of the Basque Country.

### 3.4. Mass Spectrometry Proteomic Characterization 

Protein composition was analyzed by liquid chromatography with tandem mass spectrometry (LC-MS/MS) in native ATs and DAMs. For protein extraction samples were homogenized in 8 M urea using a Precellys^®^24 homogenizer (Bertin Technologies) with 1.0-mm-diameter zirconia/silica beads (BioSpec). The homogenate was sonicated for 3 min to reduce viscosity and the crude extract was then clarified by centrifugation at 16,000× *g* for 10 min, transferred to new tubes, and stored at −20 °C. After that, protein digestion was carried out by diluting sample 5-fold with 50 mM NH_4_HCO_3_. Briefly, proteins were reduced (5 mM DTT, room temperature, 25 min), alkylated (15 mM iodoacetamide, room temperature, 30 min), and digested with trypsin (0.01 µg/µL, 37 °C, 16 h, Roche Diagnostics). Resulting peptides were desalted using C-18 Micro SpinColumns (Harvard Apparatus).

LC-MS/MS analysis was performed using a Q Exactive (Thermo Fisher Scientific, Madrid, Spain) interfaced with an Easy-nLC 1000 nanoUPLC System (Thermo Fisher Scientific, Madrid, Spain). Digested peptides were loaded onto an Acclaim PepMap100 precolumn (75 µm × 2 cm, Thermo Fisher Scientific, ES) connected to an Acclaim PepMap RSLC (50 μm × 15 cm, Thermo Fisher Scientific, ES) analytical column. Peptides were eluted directly onto the nanoES Emitter (Thermo Fisher Scientific, ES) with a 45-min linear gradient from 3% to 30% of acetonitrile in 0.1% of formic acid at a flow rate of 300 nL/min. The Q Exactive was operated in FullMS/dd-MS2 (Top10) Data-Dependent Acquisition mode. Survey scans were acquired at a resolution of 70,000 (*m/z* 200) and fragmentation spectra at 17,500 (*m/z* 200). Peptide selection was done with an isolation window of 2.0 Th and normalized collision energy of 28 was applied for peptide fragmentation. The maximum injection time was 120 ms for survey and MS/MS scans and AGC target values of 3E6 for survey scans and 5E5 for MS/MS scans were used. Raw files were processed and searched with MaxQuant1 (version 1.5.3.17) software and Andromeda2 search engine. Precursor and fragment mass tolerances were set to 4.5 and 20 ppm, respectively, and up to 2 missed cleavages were allowed. Carbamidomethylation of Cys was set as fixed modification, oxidation of Met and protein N-term acetylation as variable modifications, and a human UniProtKB-SwissProt database (version 2015_09) was used. A false discovery rate (FDR) of 0.01 for peptides and proteins and a minimum peptide length of 7 amino acids was required. Obtained results were exported into Microsoft Office Excel (Microsoft) for further analysis. 

The mass spectrometry analysis was performed in the Proteomics Core Facility-SGIKER (member of ProteoRed-ISCIII) at the University of the Basque Country.

### 3.5. Coating Preparation and Characterization 

In order to obtain a suspension appropriate for coating, 2 mg/mL milled form of the biomaterials were dissolved in 1 M acetic acid (Panreac Quimica SLU, Barcelona, Spain) under continuous stirring for 48 h at room temperature. Coatings were prepared adding 1.2 mL of solution per well to 6-well polystyrene plates and 250 µL per well and incubated at room temperature for 5 min. The same procedure was used for coating of glass coverslips in 24-well polystyrene plates. Afterwards, solutions were removed, and wells and coverslips were washed (3 × 5 min) with ultrapure Milli-Q water (Millipore, Madird, Spain), and finally, dried at room temperature for 2 h.

Coated total protein was characterized for all the DAMs by a Pierce Micro BCA Protein Assay Kit (Thermo Fisher Scientific, Madrid, Spain) following the manufacturer’s instructions. Results are shown as means ± standard deviations of *n* = 6 samples.

### 3.6. Culture of RAW-264.7 Macrophages on Decellularized Adipose Matrices (DAMs)

RAW-264.7 macrophages (10^5^ cells/mL) were cultured for 24 h at 37 °C under a 5% CO_2_ atmosphere in culture plates of six wells coated with each of the four types of extracellular matrix mentioned above (hDAM1, hDAM2, pDAM1, and pDAM2). The culture medium was Dulbecco′s modified Eagle medium (DMEM, Gibco, BRL, United Kingdom) supplemented with 10% fetal bovine serum (FBS, Gibco, BRL, United Kingdom), 1 mM l-glutamine (BioWhittaker Europe, Verviers, Belgium), penicillin (200 μg/mL, BioWhittaker Europe, Verviers, Belgium), and streptomycin (200 μg/mL, BioWhittaker Europe, Verviers, Belgium).

### 3.7. Polarization Studies of RAW-264.7 Macrophages Exposed to DAMs

#### 3.7.1. Characterization of M1 and M2 Macrophages by Flow Cytometry

M1 and M2 RAW-264.7 macrophages were quantified by immunostaining with specific antibodies and flow cytometry after exposure to the DAMs. Thus, the expression of CD80 was used as a specific marker to identify M1 macrophages and M2 macrophages were detected by the expression of CD206 and CD163. Before immunostaining, cells were detached and were incubated in 45 µL of staining buffer (PBS, 2.5% FBS Gibco, Thermo Fisher Scientific, ES, and 0.1% sodium azide, Sigma-Aldrich Corporation, St. Louis, MO, USA) with 5 µL of normal mouse serum inactivated for 15 min at 4 °C, in order to block the Fc receptors on the macrophage plasma membrane and to prevent non-specific binding of the primary antibody. Then, cells were incubated with either phycoerythrin (PE) conjugated anti-mouse CD80 antibody (2.5 µg/mL, BioLegend, San Diego, CA, USA) or fluorescein isothiocyanate (FITC) conjugated anti-mouse CD206 (2.5 µg/mL, BioLegend, San Diego, CA, USA) or Alexa Fluor 488 (A488) conjugated anti-mouse CD163 (2.5 µg/mL, BioLegend, San Diego, CA, USA) for 30 min in the dark. Labelled macrophages were then analyzed using a FACSCalibur flow cytometer. The fluorescence of these fluorochromes was excited at 488 nm and measured at 585/42 nm for PE and 490/525 for FITC and A488. The conditions for data acquisition and analysis were established using negative and positive controls with the CellQuest Program of Becton Dickinson, and these conditions were maintained in all the experiments. Each experiment was carried out three times and single representative experiments are displayed. For statistical significance, at least 10,000 cells were analyzed in each sample and the mean of the fluorescence emitted by these single cells was used.

#### 3.7.2. Morphological Studies of M1 and M2 Macrophages by Confocal Microscopy

For confocal microscopy studies, RAW-264.7 macrophages were cultured on circular glass coverslips coated with each of the four DAMs under the above-mentioned culture conditions. Cells were fixed with 3.7% paraformaldehyde (Sigma-Aldrich Corporation, St. Louis, MO, USA) in PBS for 10 min, washed with PBS, and permeabilized with 0.1% Triton X-100 (Sigma-Aldrich Corporation, St. Louis, MO, USA) for 5 min. The samples were then washed with PBS and preincubated with PBS containing 1% BSA (Sigma-Aldrich Corporation, St. Louis, MO, USA) for 30 min to prevent non-specific binding. Samples were incubated in 1 mL of staining buffer with either PE conjugated anti-mouse CD80 antibody (2.5 µg/mL, BioLegend, San Diego, California) or FITC conjugated anti-mouse CD206 (2.5 µg/mL, BioLegend, San Diego, California) or A488 conjugated anti-mouse CD163 (2.5 µg/mL, BioLegend, San Diego, California) for 30 min at 4 °C in the dark. Samples were then washed with PBS and the cell nuclei were stained with DAPI (4′-6-diamidino-2′-phenylindole, 3 μM in PBS, Molecular Probes) for 5 min. Samples were examined by a LEICA SP2 Confocal Laser Scanning Microscope. The fluorescence of FITC, PE, and A488 was excited at 488 nm and the emitted fluorescence was measured at 491–586 nm for FITC and 575–675 nm for PE and A488. DAPI fluorescence was excited at 405 nm and measured at 420–480 nm.

#### 3.7.3. Detection of TNF-α as an Inflammatory Cytokine

The amount of TNF-α secreted by RAW-264.7 macrophages under the different conditions was quantified in the culture medium by the enzyme-linked immunosorbent assay (ELISA, Gen-Probe, Diaclone, Besançon, France) according to the manufacturer’s instructions.

#### 3.7.4. Intracellular Reactive Oxygen Species (ROS) Content Measured by Flow Cytometry

After exposure to the DAMs, RAW-264.7 macrophages were detached and cell suspensions were incubated with 100 μM of 2′,7′-dichlorofluorescein diacetate (DCFH/DA, Serva, Heidelberg, Germany) during 30 min at 37 °C. DCF fluorescence depends directly on the intracellular content of reactive oxygen species (ROS) and was measured in a FACScalibur Becton Dickinson flow cytometer with a 530/30 filter, exciting the sample at 488 nm. In each sample, 10^4^ cells were analyzed.

### 3.8. Immunocompetence Studies of RAW-264.7 Macrophages Exposed to DAMs and Infected with Candida albicans

#### 3.8.1. *Candida albicans* Strain

The *C. albican* strain used in this study was the CAF2-dTOM2 derived from SC5314 expressing a red fluorescent protein (RFP) called RFP-*Candida albicans* [73], kindly provided by Dr. D. Prieto and Dr. J. Pla. This red fluorescent strain was synthesized by codon optimization of the DsRed-derived RFP dTomato gene [74] using the tetracycline-dependent integrative plasmid pNIM1R. Expression in *C. albicans* was also detectable in SD plates as pink-reddish-colored colonies after two days of growth [75]. The yeast strain was short-term stored at 4 °C and grown at 37 °C in YPD medium (2% glucose, 2% peptone, 1% yeast extract) plus amino acids and chloramphenicol (10 µg/mL) for 48 h.

#### 3.8.2. Phagocytosis of *Candida albicans* Evaluated by Flow Cytometry and Confocal Microscopy

RAW-264.7 macrophages were seeded during 24 h either onto 6-well tissue culture plates or onto sterile 12-mm-diameter round glass coverslips coated with DAMs (hDAM1, hDAM2, pDAM1, and pDAM2) under the above-mentioned culture conditions at a density of 10^5^ cells per mL at 37 °C under a 5% CO_2_ atmosphere. Fungal cells (RFP-*Candida albicans)* were collected and washed with PBS and adjusted to the desired cell density for macrophage infection. Two macrophage:fungus ratios were employed, referred to as multiplicity of infection (MOI) MOI 1 and MOI 5. Controls with macrophages without *C. albicans* infection were carried out in parallel. After 45 min of interaction at 37 °C under a 5% CO_2_ atmosphere, RFP-*Candida albicans* phagocytosis by RAW-264.7 macrophages was analyzed by flow cytometry and confocal microscopy. Non-ingested and unbound *Candida* cells were removed by washing three times with ice-cold PBS. The macrophages and bound/ingested yeast cells were dislodged by scraping the flask with macrophage interaction rubber scrapers in ice-cold PBS to be further analyzed in a FACScalibur Becton Dickinson flow cytometer. *C. albicans* was excited at 488 nm and measured with 530/30 and 585/42 band pass filters, respectively, using a FACScalibur Becton Dickinson flow cytometer. For confocal microscopy studies, the cells were fixed with 3.7% paraformaldehyde in PBS and the cell nuclei were stained with DAPI (4′-6-diamidino-2′-phenylindole, 3 M in PBS, Molecular Probes) for 5 min, using a Leica SP2 Confocal Laser Scanning Microscope. The fluorescence of RFP-expressing *C. albicans* was excited at 488 nm and measured at 584/663 nm. DAPI fluorescence was excited at 405 nm and measured at 409/468 nm. In order to quantify RFP-*Candida albicans* phagocytosis by RAW-264.7 macrophages, at least 10 fields were observed in the different samples and 500 cells were counted in each condition.

### 3.9. Statistics

Data are expressed as means ± standard deviations of a representative of three experiments carried out in triplicate. Statistical analysis was performed using the 22nd version of Statistical Package for the Social Sciences (SPSS). Statistical comparisons were made by analysis of variance (ANOVA). Scheffé test was used for post hoc evaluations of differences among groups. In all the statistical evaluations, *p* ˂ 0.05 was considered as statistically significant.

## 4. Conclusions

In this work, we characterized four extracellular matrices from human and porcine adipose tissue, decellularized by enzymatic and chemical methods, in order to evaluate their possible use to prepare bio-scaffolds for regenerative medicine. This study demonstrates that none of these matrices induces a macrophage proinflammatory response, but a reparative behavior was mainly observed after exposure to the porcine matrices. The presence of differentially preserved proteins after decellularization relies on both the tissue origin and the method employed. For the first time, we evidenced that ECM-specific proteins play a key role in the macrophage response to these natural biomaterials. Moreover, these findings highlight the sensitivity of the fungal phagocytosis assay to assess the response of macrophages to the environment and encourage further research on these matrices for injured tissue repair, where the macrophage function is essential for tissue regeneration.

## Figures and Tables

**Table 1:** Remnant DNA quantity (ng DNA/mg DAM dry weight) after tissue decellularization in all assayed decellularized adipose matrices (DAMs).

DAM	ng DNA/mg DAM	AVERAGE	SD
hDAM1	2.28	2.38	0.16
	2.29		
	2.56		
pDAM1	0.90	0.89	0.02
	0.89		
	0.87		
hDAM2	2.81	3.71	1.2
	3.25		
	5.08		
pDAM2	26.10	24.8	2.05
	25.87		
	22.43		

**Table 2:** Remnant lipid quantity (µg/mg DAM) after tissue decellularization in all assayed DAMs by lipid class and percentage (*w/w*) in respect to the DAM dry weight. Values are reported as the mean ± standard deviation of *n* = 3 samples. Abbreviations: LPC: lysophosphatidylcholine, LPE: lysophosphosphatidyl ethanolamine, PC: phosphatidylcholine, SM: sphingomyelin, SPH: sphingolipid, Cer: ceramide, DG: diacylglycerol, TG: Triacylglycerol, ChE: cholesterol ester.

	µg Lipid/mg DAM							
	hDAM1		pDAM1		hDAM2		pDAM2	
Lipid Molecule	Average	SD	Average	SD	Average	SD	Average	SD
LPC	0.0	0.0	0.1	0.0	0.0	0.0	0.0	0.0
LPE	0.0	0.0	0.0	0.0	0.0	0.0	0.0	0.0
PC	0.0	0.0	0.2	0.1	0.0	0.0	0.0	0.0
SM	0.1	0.0	0.3	0.3	0.0	0.0	0.0	0.0
SPH	0.0	0.0	0.0	0.0	0.0	0.0	0.0	0.0
Cer	0.0	0.0	0.0	0.0	0.0	0.0	0.0	0.0
DG	0.0	0.0	0.0	0.0	0.0	0.0	0.5	0.0
TG	0.4	0.0	0.8	0.3	5.7	1.5	69.0	2.0
ChE	0.2	0.1	1.4	0.3	Nd	nd	nd	nd
Total	0.8	0.1	3.0	1.0	5.8	1.5	69.6	2.1
% (*w/w*)	0.1	0.0	0.3	0.1	0.6	0.2	7.0	0.2

**Table 3:** Coated total protein on polystyrene and glass coverslip for all the DAMs (hDAM1, pDAM1, hDAM2, pDAM2).

DAM	Coated Protein (µg/cm^2^)			
	Polystyrene		Glass Coverslip	
	Average	SD	Average	SD
hDAM1	1.31	0.16	1.06	0.20
hDAM2	1.54	0.16	0.79	0.04
pDAM1	0.94	0.17	0.90	0.21
pDAM2	1.23	0.11	0.89	0.23

**Table 4:** Effects of hDAM1, hDAM2, pDAM1, and pDAM2 on specific markers of M1 and M2 phenotypes of RAW-264.7 macrophages exposed for 24 h to these matrices.

Sample	M1-Phenotype			M2-Phenotype		
	CD80	TNFα	High ROS	CD163	CD206	Low ROS
*hDAM1*	↓	=	=	=	=	↓↓
*hDAM2*	=	=	↓↓	=	=	↑↑
*pDAM1*	=	↓↓	↓↓	↑↑	=	↑↑
*pDAM2*	=	↓	↓↓	↑↑	↑	↑↑

## Data Availability

Data is contained within the article.

## Supplementary Materials

The following are available online at https://www.mdpi.com/article/10.3390/ijms22083847/s1, Table S1: Human adipose tissue ECM proteins, and Table S2: Porcine adipose tissue ECM proteins.
